# Clinical application of operative hysteroscopy in treatment of complex hydrosalpinx prior to IVF

**Published:** 2015-05

**Authors:** Hong-Chu Bao, Mei-Mei Wang, Xin-Rong Wang, Wen-Juan Wang, Cui-Fang Hao

**Affiliations:** *Reproductive Medicine Center. Yantai Yuhuangding Hospital, Qingdao University, Yantai, China.*

**Keywords:** *Hydrosalpinx*, *Infertility*, *Hysteroscopy*, *Tubal occlusion*

## Abstract

**Background:**

In vitro fertilization and embryo transfer (IVF-ET) is the best option for patients with hydrosalpinx. However, if hydrosalpinges is not pre-treated, the therapeutic outcomes of IVF-ET would be compromised.

**Objective:**

This study aims to investigate the safety and effects of operative hysteroscopy in the treatment of patients with hydrosalpinx prior to IVF-ET, who were not indicated for laparotomy due to extensive pelvic adhesion.

**Materials and Methods:**

The study analyses retrospectively data from 10 women with hydrosalpinx, who were unable to undergo laparotomy due to extensive pelvic adhesion and treated by operative hysteroscopy prior to IVF-ET, and was assessed the effects and safety of the procedure.

**Results:**

Postoperative Hystero-salpingography demonstrated complete tubal occlusion of the diseased side in all cases. Being applied with IVF-ET for fertility after their hysteroscopy operation, 5 out of 10 patients acquired clinical pregnancy.

**Conclusion:**

Hysteroscopic tubal occlusion of the proximal part of the hydrosalpinx can effectively prevent the hydrops backflow to endometrial cavity and benefit subsequent implantation in the course of assisted reproduction without significant complications.

## Introduction

Hydrosalpinx is one of the common infertility factors in female infertility. The primary selection of fertility problem for patients with hydrosalpinx is in vitro fertilization and embryo transfer (IVF-ET). However, such patients will harvest an inferior treatment effect (1) without undergoing pretreatment prior to IVF-ET. The specific mechanism is still undetermined, primarily because the fluid secreted by epithelial cells in hydrosalpinx lumen will reflux to uterine cavity via internal orifice of uterine tube once it forms sufficient pressure, and thus “mechanically scours” the embryo; the embryo receives a toxic action and its development is affected; the receptivity of endometrium is damaged and therefore the embryo implantation and pregnancy rates decreased and the abortion rate increased (2, 3). 

Consequently, pretreatment is critical for severely diseased hydrosalpinx prior to IVF-ET-based fertility. There are many approaches for treating hydrosalpinx, which have their own advantages and disadvantages. However, for some patients who cannot undergo abdominal surgery due to severe pelvic adhesion, operative laparoscopy or tubal laparotomy may induce major complications such as damages to digestive and urinary systems. This is what we term as complex hydrosalpinx. Since 2006, we have observed 10 cases with complex hydrosalpinx, and herein summarize their clinical characteristics and IVF-ET outcomes.

## Materials and methods

**Subjects**

In this retrospective study, 419 medical records of women with hydrosalpinx underwent surgical laparoscopic procedures performed by one surgeon in Yantai Yuhuangding Hospital from May 2006 to June 2011were reviewed. Study protocol was approved by Yantai Yuhuangding Hospital Ethics committee. Among these women, 10 cases could not be performed laparoscopic procedure because of extensive pelvic adhesion (either because of preoperative evaluation on severe pelvic adhesion or have to stop laparoscopy due to extremely severe pelvic adhesion). The total of 10 women with hydrosalpinx underwent the fulguration of internal orifice of uterine tube under hysteroscopy due to severe pelvic adhesion. The institutional review board of Yantai Yuhuangding Hospital approved this study, and all patients gave their informed consent for the inclusion in the study. The results of vaginal ultrasonic examination performed on them showed that there were irregular echo-free areas outside ovary, irregular vaginal discharge, and significant shrinkage or even disappearance of irregular low-echo areas outside bilateral ovaries after vaginal discharge, and also suggested existence of hydrosalpinx fluid reflux. The characteristics of each woman before and after operative hysteroscopy are shown in table I.

**Surgical method**

Under intravenous or epidural anesthesia, a monopolar roller ball electrode (size: 3 mm) is clung to the internal orifice of affected-side fallopian via hysteroscopy. The electrocoagulation was performed with power of 40-60W for 5~10 sec at each side to form a deep yellow scabbed plaques. The electrical heat energy was utilized to degenerate the internal orifice tissue of diseased fallopian so as to scar the electro coagulated tissue. The contracting and even occluding of the scarred tissue prevented the hydrosalpinx fluid reflux to uterine cavity (see attached pictures). a vaginal ultrasonic examination were be taken on the 2^nd^ or 3^rd^ day after surgery procedure. 

If the examination results suggest the existence of hydrosalpinx fluid (uterine distention fluid might flow into the tubes during hysteroscopic procedure), hydrosalpinx aspiration and anhydrous alcohol sclerotherapy were applied under the guidance of ultrasound so as to abate the impact of fluid reflux on the formation of scare of fulguration wound during the first few days after the performance. In one or two months, hysterosalpingography was performed to confirm postoperative obstruction blockade. 

**Assisted reproductive method**

There was a standard long protocol or ovarian mild stimulation protocol to be adopted for IVF-ET-based fertility according to patient’s various ovarian functions. For patients whose ovarian functions were normal, a standard long protocol were adopted, namely pituitary down-regulation at mid-luteal phase before ovulation induction; for those with ovarian hypofunction, an ovarian mild stimulation protocol were adopted, namely 2~3 cycles of hormone replacement therapy before ovulation induction by oral Clomiphene combined with intramuscular injection of human menopausal gonaclotxopin (HMG).

## Results

**The safety and effects of operative hysteroscopy for treating hydrosalpinx**

As shown in table I, the hysteroscopic electrocoagulation of internal orifice of uterine tube performed on patients with complex hydrosalpinx could effectively prevent hydrosalpinx fluid reflux and induce no significant complications after surgery. Of 10 women, 4 were found not suitable for either operative laparoscopy or laparotomy due to extremely severe pelvic adhesion at the beginning of laparoscopic procedure and therefore switched to hysteroscopic surgery; the remaining 6 were advised to undergo operative hysteroscopy because the preoperative evaluation suggested that they had severe pelvic adhesion. 

In order to increase the probability of the obstruction blockade by scar formed at postoperative electro coagulated position, a vaginal ultrasonic examination can be performed on the 2^nd^ or 3^rd^ day after surgery to see if there is any hydrosalpinx fluid in the diseased tube. If it existing, an ultrasound-guided hydrosalpinx aspiration and an anhydrous alcohol sclerotherapy can be used so as to abate the impact of fluid reflux on the scar formation of fulguration wound. After anhydrous alcohol sclerotherapy, some mucosa of fallopian may be damaged and hence lose its secretion function so as to disable or delay the relapse of hydrosalpinx. However, some patients still had repeated relapses of hydrosalpinx even though they received hydrosalpinx aspiration and anhydrous alcohol sclerotherapy previously; thus they would require several times puncture. 

According to the conditions of disappearance of postoperative vaginal discharge and postoperative hysterosalpingography, status of hydrosalpinx fluid reflux was confirmed. The postoperative HSG demonstrated complete tubal occlusion of the diseased side in all cases. Patient No.3 underwent second-look office hysteroscopy due to repeated failures of IVF-ET, and the results revealed bilateral salpingemphraxis and no significant corneal lesions at the site of hysteroscopic occlusion.

**Fertility effect of operative hysteroscopy in the treatment of hydrosalpinx**

Patients got individualized protocols of IVF-ET-based fertility treatment depending on their ovarian functions after they underwent operative hysteroscopy, and the postoperative reproductive effect was as shown in table II. The patients were assigned with either a standard long protocol or an ovarian mild stimulation protocol for IVF-ET depending on their ovarian functions after operative hysteroscopy. 5 out of 10 patients acquired clinical pregnancy. 

Patient No.9 aborted at midtrimester of pregnancy due to previous conization of cervix; patient No.6 experienced intestinal obstruction at medium-term and received expectant treatment and was succeeded in full-term normal delivery finally; patient No.4 was performed caesarean at week 38 due to placenta praevia when twin boys were born. And during caesarean surgery, the bilateral fallopian of the patient were excised because of their sausage-like thickening appearance. The remained 2 cases had a stable pregnancy course.

**Table I:** Characteristics of patients before and after operative hysteroscopy

**No**	**Age**	**HSG diagnosis**	**Basic FSH**	**Preoperative vaginal discharge**	**Causes of abdominal adhesion**	**Postoperative vaginal discharge**	**Postoperative complication**	**Postperative HSG diagnosis on relapse of hydrosalpinx**
1	38	Bilateral hydrosalpinx	8.6	Yes	1 laparotomy	No	No	No
2	31	Bilateral hydrosalpinx	7.9	Yes	T.B. peritonitis	No	No	No
3	32	Bilateral hydrosalpinx	8.1	Yes	appendectomy	No	No	No
4	29	Bilateral hydrosalpinx	5.6	Yes	1 pelvic infection	No	No	No
5	40	Bilateral hydrosalpinx	15.2	Yes	2 pelvic surgeries	No	No	No
6	34	Bilateral hydrosalpinx	25.5	Yes	4 laparotomies for endometriosis	No	No	No
7	37	Bilateral hydrosalpinx	20.4	Yes	2 pelvic laparotomies	No	No	No
8	31	Bilateral hydrosalpinx	7.2	Yes	2 pelvic laparotomies	No	No	No
9	36	Bilateral hydrosalpinx	7.5	Yes	1 laparotomy	No	No	No
10	31	Bilateral hydrosalpinx	6.4	Yes	1 surgery for splenic rupture	No	No	No

HSG: Hysterosalpingography

* (mIU/ml)

**Table II:** Results of IVF-ET-based Fertility after Operative Hysteroscopy

**No**	**Age**	**IVF-ET protocol**	**Number of cycle**	**Occurrence of hydrosalpinx during ovulation induction**	**Pregnant** **Yes/No**	**Pregnancy outcome**	**Pregnancy complication**
1	38	Standard long down-regulation protocol	1	No	Yes	Full-term pregnancy and successful delivery of singleton	
2	31	Standard long down-regulation protocol	1	No	No	None	
3	32	Standard long down-regulation and mild stimulation protocol	5	No	Biochemical pregnancy abortion	None	placenta praevia
4	29	Standard long down-regulation protocol	1	Yes	Yes	Cesarean birth of 38-week twins	
5	40	Mild stimulation protocol	2	No	No	None	
6	34	Mild stimulation protocol	3	Yes	Yes	Successful delivery of singleton	Intestinal obstruction successfully treated by conservative therapy
7	37	Mild stimulation protocol	3	Yes	No	None	
8	31	Standard long down-regulation protocol	1	Yes	No	None	
9	36	Standard long down-regulation protocol	1	Yes	Yes	Abortion after 16 weeks	Cervical incompetence
10	31	Standard long down-regulation protocol	1	Yes	Yes	Still in singleton pregnancy	None

**Figure 1:**
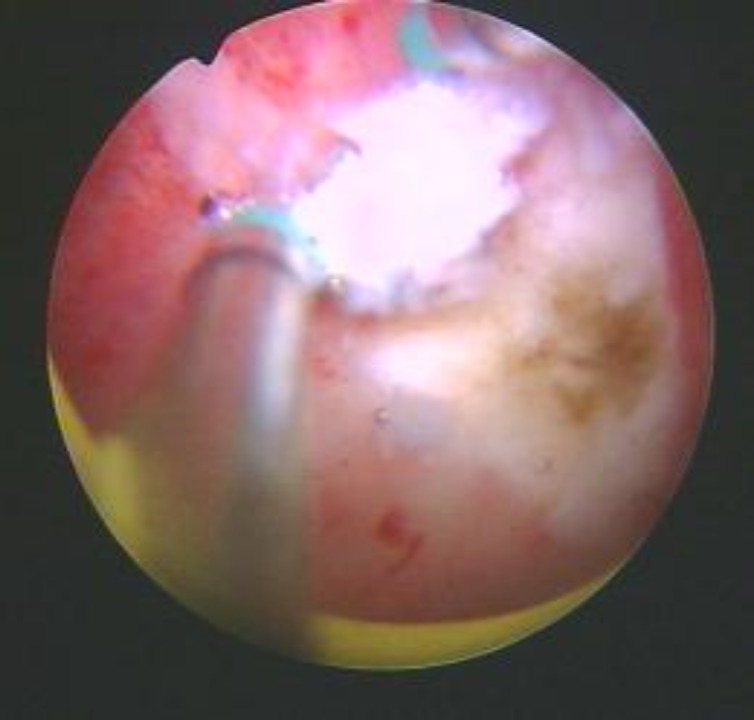
Internal orifice of uterine tube after electrocoagulation

## Discussion

When the volume of hydrosalpinx fluid is accumulated to a certain extent, it will reflux to uterine cavity via internal orifice of uterine tube to induce paroxysmalvaginal discharge. Afterwards, hydrosalpinx may shrink in volume and ultrasonic re-examination can demonstrate the smaller or disappeared low- echo area outside ovary compared to previous examination. The approaches for hydrosalpinx pretreatment before IVF-ET consist of different types including salpingectomy, salpingostomy and proximal tubal obstruction surgery, and each of them is able to increase the clinical pregnancy and implantation rates as well as decreases the rate of ectopic pregnancy and abortion of IVF-ET in certain extent. For patients with hydrosalpinx, the results of intraoperative exploration, such as pelvic adhesion degree and nature, diameter of tubal ampulla, condition of damaged tubal mocusa and tubal wall thickness should be considered and regarded as the key factors in evaluation of surgical effect.

 Although some investigators consider that salpingectomy doesn’t affect ovarian response during IVF-ET, evidences suggest that conventional salpingectomy may affect ovarian blood supply and follicular development, and hence reduce the number of retrieved oocytes (4, 5). Therefore, it should only be applied to patients with strict indication; that is, the significant dilation of tubal lumen shown on ultrasonic examination. In laparoscopic surgery, ultrasound scalpel or bipolar coagulation should be used as far as possible to isolate mesosalpinx tissue, while unipolar current should be avoided for reducing damages of intra-mesosalpinx neurovascular nets and avoiding the reduction of ovary blood supply. 

To patients with slightly diseased tubal fimbriae, salpingoplasty (if possible) may be applied in order to acquire expected natural pregnancy; however, hydrosalpinx may easily recur after salpingostomy and require repeated surgery (6, 7). To patients with hydrosalpinx affecting fertility severely, salpingectomy can be applied in combination with proximal tubal ligation to prevent hydrosalpinx fluid reflux to uterine cavity and increase the probability of conception. In a randomized controlled study enrolling 115 patients with hydrosalpinx, the rates of ongoing pregnancy were 46% and 34% in patients either receiving salpingectomy or proximal tubal ligation prior to IVF respectively. These rates were greatly increased compared the patients without surgical intervention (6.6%) (8). 

Such approaches are suitable for patients who cannot undergo salpingectomy due to severe pelvic adhesion and have the advantages of relatively simpler procedures and fewer damages to patients. However, fimbriaeneostomy may cause more relapse of hydrosalpinx if proximal tubal ligation is adopted, and fluid may reoccur in tubal lumen to induce large tubal cyst with torsion later (9). To patients with hydrosalpinx who cannot undergo laparoscopic surgery due to excessive fatness and extensive abdominal adhesion, a newly developed approach can be applied. This procedure requires the obstruction of the interstitial portion of diseased tubal with microemboli placement under hysteroscope to block the reflux of hydrosalpinx fluid. It has been wildly utilized in clinic based on the benefits of this procedure such as simple operation, high successful rate, rapid rehabilitation, minor injury, no requirement of general anesthesia as well as no severe complications happening like intestinal canals obstruction and blood vessels damage which are commonly occurred in operative laparoscopy or laparotomy (10, 11). It can also be applied to patients with unilateral hydrosalpinx. Omurtag et al reported that a patient with left-side hydrosalpinx acquired natural pregnancy after proximal uterine tube was placed with microemboli (12). 

As like tubal ligation, this procedure can also induce the large tubal cyst, which may distort and induce the complications such as less retrieved oocytes. 

Accordingly, it is just indicated for the patients with contraindications of abdominal surgery. In addition, it also requires special hysteroscope equipment and obstruction materials and cannot be performed in most regions. Proximal tubal obstruction surgery can also be performed via uterine cervix under the guidance of X-ray to create proximal tubal obstruction for preventing hydrosalpinx fluid reflux. During the procedure, a micro spring coil is placed at tubal interstitial portion or tubal isthmus through the microtubule to form mechanical obstruction. But the microemboli may theoretically change the internal environment of uterine cavity and even induce uterine contractility during pregnancy due to long-term retention of foreign materials in uterus (13). Consequently the evaluation of its safety and effects will still require the long- term observation and large-sample studies. 

Ultrasound-guided hydrosalpinx puncture and aspiration is another pretreatment method prior to IVF-ET, mostly is performed at ovum pick-up time, especially applicable for patients who are not found with hydrosalpinx before IVF-ET (14). This method has such advantages as less damages, convenient operation and wide acceptance by patients. However, hydrosalpinx may relapse in a few days or hours (15, 16). And the finding of multiple adhesions at laparoscopy after sclerotherapy was described by Shokeir (17).

Hysteroscopic fulguration of internal orifice of fallopian tubes is designed to degenerate internal orifice tissue of diseased tube by electric heat energy to form tissue scar so as to prevent hydrosalpinx fluid reflux to uterine cavity, helping embryo’s development and implantation and maximizing the protection of intra-mesosalpinx blood vessels and nerves theoretically. Experiments in vitro suggested that coagulation of tubal internal orifice at a hysteroscopic unipolar coagulation power of 50w within duration of 20s doesn’t damage other fractions of endometrium and uterine serosa layer (18). However, this procedure is just an exploratory therapy for such patients. Although these patients showed no episodic vaginal discharge after surgery, in part of them the fluid didn’t disappear after surgery. Instead, it remained in fallopian tubes and grew bigger to possibly induce tubal rupture or torsion. Hence, this approach should only be applied to patients who cannot undergo abdominal surgery due to severe pelvic adhesion. Besides, the ultrasound-guided hydrosalpinx aspiration and anhydrous alcohol sclerotherapy after hysteroscopic operation may aid to reduce the relapse of hydrosalpinx and avoid tubal rupture. In addition, once patient with extensive pelvic adhesion is pregnant, she may suffer from the complications of intestinal obstruction induced by distorted intestinal canal position with the expansion of uterus during pregnancy, therefore, the close observation of digestive symptoms is necessary in this period (19, 20).

In conclusion, this preliminary study demonstrates hysteroscopic tubal occlusion of the proximal part of the hydrosalpinx can effectively prevent the hydrosalpinx fluid backflow to uterus cavity and benefit the embryo implantation in the course of IVF-ET without significant complications. The procedure is a safe, effective, fast, and easy approach. And it can be carried out in out- patient department under local paracervical block. However, the surgery procedure is restricted only to the patients who are unable to accept the laparotomy or operative laparoscopy due to excessive pelvic adhesion. Further large sample-sized studies are required to test its impact on the implantation rate and clinical outcome. Once pregnancy undergoing, it requires close observations for any surgical complications caused by pelvic adhesion such as intestinal obstruction, and theoretical risk of cornual rupture should be taken into considerations in labor concerning the thermal spread.
